# The role of HMGB1-RAGE axis in migration and invasion of hepatocellular carcinoma cell lines

**DOI:** 10.1007/s11010-014-1978-6

**Published:** 2014-02-09

**Authors:** Ruo-Chan Chen, Pan-Pan Yi, Rong-Rong Zhou, Mei-Fang Xiao, Ze-Bing Huang, Dao-Lin Tang, Yan Huang, Xue-Gong Fan

**Affiliations:** 1 Department of Infectious Diseases, Key Laboratory of Viral Hepatitis of Hunan, Xiangya Hospital, Central South University, Changsha, 410008 China; 2 Department of Surgery, Hillman Cancer Center, University of Pittsburgh Cancer Institute, Pittsburgh, PA 15219 USA

**Keywords:** HMGB1, RAGE, Hepatocellular carcinoma, Invasion, Migration

## Abstract

High mobility group protein box1 (HMGB1) and its receptor—receptor for advanced glycation end products (RAGE) are pivotal factors in the development and progression of many types of tumor, but the role of HMGB1-RAGE axis in hepatocellular carcinoma (HCC) especially its effects on metastasis and recurrence remains obscure. Here, we report the role of HMGB1-RAGE axis in the biological behaviors of HCC cell lines and the underlying molecular mechanism. We show that the expressions of HMGB1, RAGE, and extracellular HMGB1 increase consistently according to cell metastasis potentials, while the concentration of soluble form of RAGE (sRAGE) is inversely related to metastasis potential of HCC cells. Furthermore, our data show that rhHMGB1 promotes cellular proliferation, migration, and invasion, and increases the level of nuclear factor kappa B (NF-κB), while administrations of HMGB1-siRNA, RAGE-siRNA, anti-HMGB1 neutralizing antibody, anti-RAGE neutralizing antibody, and sRAGE inhibit cellular proliferation, migration, and invasion. Moreover, we also demonstrate that the expression of NF-кB is inhibited by knockdown of HMGB1 or RAGE. Collectively, these data demonstrate that HMGB1 activates RAGE signaling pathways and induces NF-кB activation to promote cellular proliferation, invasion, and metastasis, in HCC cell lines. Taken together, HMGB1-RAGE axis may become a potential target in HCC therapy.

## Introduction

Hepatocellular carcinoma (HCC) is one of the most common malignancies around the world, especially in Asia and Africa. Despite the progress attained in molecular biology and cancer therapy recent years, the remote prognosis of HCC remains dismal, which was mainly attributed to the high incidence of distant metastasis, therapy resistance, and local recurrence [1]. However, the underlying molecular mechanism responsible for invasion and metastasis of HCC still remains obscure. Therefore, it is of great significance to make further study about the molecular mechanism of metastasis and invasion of HCC.

High mobility group protein box1 (HMGB1) is a nonhistone chromatin-binding protein. The function of HMGB1 is complicated and related to its cellular localization. In the nucleus, HMGB1 binds with DNA and serves as a structural component. Nevertheless, it can be released into the extracellular environment during necrosis or in response to certain stimulations, such as hypoxia and endotoxin [2]. Through the binding with high affinity to several receptors, extracellular HMGB1 plays a critical role in the development of various diseases, such as vascular disease, diabetes, cancer, and neurodegeneration [3]. Receptor for advanced glycation end products (RAGE), which is a member of the immunoglobulin superfamily of cell surface molecules, is a central cell surface receptor for HMGB1 [4–6]. Experimental data have shown that through the activation of various signaling pathways such as NF-кB, Racl/Cdc42, MAPKs, PI3K/Akt, ERK1/2, SrcK, and HMGB1-RAGE interaction is deeply involved in tumor growth, migration, and metastases [4, 7]. HMGB1 is highly expressed in many types of tumors, such as pancreatic cancer, colon cancer, and melanoma [8]. Previous study found that HMGB1 and RAGE can modulate the proliferation of HCC cells and induce apoptosis, indicating that HMBG1-RAGE axis may play a significant role in the development of HCC [9, 10].

NF-κB is a dimeric protein widespread in the cytoplasm. Through the gene products and downstream signaling pathways, NF-κB participated in numerous pathological processes, such as inflammation, immune response, apoptosis, tumor and cell cycle regulation, cell differentiation, and so on [11–13]. Activated NF-κB is highly expressed in inflammation-related HCC and serves as a bridge from chronic inflammation to carcinogenesis [14–16]. Accumulating evidences showed that HMGB1 can promote the activation of NF-κB through binding with RAGE in macrophages and oral squamous cell carcinoma [17, 18].

However, the biological function of HMGB1-RAGE axis in the invasion of HCC is yet not fully understood. In this study, we evaluate that whether HMGB1 can induce NF-κB activation through the interaction with RAGE and subsequently contribute to tumor invasiveness in HCC cells.

## Materials and methods

### Cell culture and regents

Three human HCC cell lines with unique metastatic characteristics were investigated. Hep3B cell line with the lowest metastasis potential [19] was purchased from Cell Bank of the Chinese Academy of Sciences (Shanghai, China); MHCC97L cell line with the moderate metastasis potential and HCCLM3 cell line with the highest metastasis potential were kindly obtained from the Liver Cancer Institute of Fudan University (Shanghai, China) [20, 21]. All cell lines were maintained in DMEM medium (Gibico), supplemented with 10 % fetal bovine serum (FBS, Gibico), penicillin (100 U/ml), and streptomycin (100 μg/ml). Cells were incubated at 37 °C with 5 % CO_2_, and were harvested with 0.25 % trypsin/EDTA (Gibico) in their logarithmic growth phase, washed with PBS, and resuspended in new media. The reagents used were as follows: Recombinant human HMGB1 (rhHMGB1) and recombinant human RAGE/Fc chimera, homologs to soluble form of RAGE (sRAGE), utilized as ex-RAGE were purchased from R&D systems (Inc., Minneapolis, MN, USA). The antibodies used were as follows: anti-HMGB1 neutralizing antibody, anti-RAGE neutralizing antibody (Abcam), anti-GAPDH (Cell Signal Technology), anti-NF-κB P50, anti-NF-κB P65, and goat IgG (Santa Cruz).

### ELISA

Hep3B, MHCC97L, and HCCLM3 cells were plated in 6-well plates at a density of 1 × 10^4^ cells/well. After incubation for 48 h, supernatants were collected to detect the release levels of extracellular HMGB1 and sRAGE by ELISA (R&D system), according to the manufacturer’s instructions.

### HMGB1 and RAGE knockdown by siRNA

HCCLM3 cells were seeded in 6-well plates at a density of 1 × 10^5^ cells/well to achieve a confluence of 80–90 % overnight. Then, HMGB1-siRNA, RAGE-siRNA, and negative control siRNA (GenePharma RNAi Company, Shanghai, China) were transfected into cells, respectively, using transfection reagent (lipofectamine 2000, Invitrogen) according to the manufacturer’s instructions.

### Cell viability assay

Both trypan blue exclusion and MTT methods were employed to assess with the cell viability of HCCLM3 cells. After incubation for 24–72 h, 0.5 mg/ml MTT was added to fresh medium, then cells were incubated for an additional 4 h. Afterward, the formazan crystals were dissolved in 150 μl dimethyl sulphoxide (DMSO, Sigma-Aldrich) and measured optical density (OD) at double wavelength (405, 630 nm) with Multiskan Ascent System (Thermo).

### RT-PCR

Total RNA was extracted with Trizol (Invitrogen, USA) from cells. 1 μg of RNA was reverse transcribed into cDNA using the Premium First Strand cDNA Synthesis Kit (Fermentas). PCR amplifications followed an initial denaturation at 95 °C for 5 min, 30 cycles consisting of 95 °C for 30 s, 65 °C for 30 s, and 72 °C for 1 min, and finally stored at 72 °C for 7 min. PCR products were separated by electrophoresis using 2 % agarose gels. PCR band intensities were compared to β-actin using the Image J (National Institutes of Health, USA). Quantity RT-PCR reactions were performed with a Applied Biosystem 7500 Real-Time PCR System at 50 °C for 2 min, 95°C for 2 min, 40 cycles of 95 °C for 15 s, 60 °C for 32 s. 20 μl amplification reactions were carried out containing 10 μl 2 × Platinum SYPR Green qPCR SuperMix-UDG (Invitrogen), 1 μl primers, and 1 μl cDNA.

### Western blotting

Whole cell protein was extracted with Radio-Immunoprecipitation Assay (RIPA, Beyotime Institute of Biotechnology, China). Protein samples were separated by 12.5 % SDS-PAGE and transferred to PVDF membranes (0.45 μm, Millipore) and blocked for 1 h with 5 % bovine serum albumin (BSA), 0.1 % Tween-20 in TBS. The membranes were then washed and incubated separately with the following primary antibodies: anti-HMGB1 (1 μg/ml), anti-RAGE (2 μg/ml), and anti-GAPDH. After 1 h at room temperature, the goat anti-rabbit or goat anti-mouse IgG secondary antibodies (1:5,000 dilution)were used to incubate another an hour. After washing, the immunoreactive complexes were detected with ECL chemiluminescence reagents (Invitrogen). ImageJ was used to measure the density of the bands imaged by X-ray films.

### Cell invasion assay

Cell invasion was studied using Corning Transwell polycarbonate membrane inserts with 24 pores (pore size 8.0 μm, membrane diameter 6.5 mm). The inserts were set into 24-well plates to form two champers, and the upper chambers were precoated with Matrigel Basement Membrane Matrix (BD Biosciences-Discovery Labware). HCCLM3 cells in the logarithmic growth phase were trypsinized and resuspended in nonserum containing DMEM media. Cells in 300 μl DMEM media with a density of 5 × 10^3^ cells/ml were plated in the upper chambers. 500 μl DMEM medium with 10 % FBS was used as a chemoattractant in the lower chamber. Cell invasion was allowed to progress for 24 h at 37 °C with 5 % CO_2_. Afterward, the Matrix gel and cells on the top membrane surface were removed with a cotton swab. Transwell membranes were then stained with crystal violet, and the light microscopy was used to photograph the cells that migrated through the membrane to the lower surface. The migrating cells were quantified by dissolving the purple crystals on the membranes in 500 μl 10 % acetic acid, and measuring their OD values at 570 nm by Multiskan Ascent.

### Wound healing assay

Wound healing assay was performed to evaluate cell motility of HCCLM3 cells. Every 0.5 cm a line across the hole was drawn on the basal sides of 12-well plates. HCCLM3 cells were trypsinized, resuspended in nonserum containing DMEM medium. A total of 5 × 10^5^ cells were plated into the holes for 24 h to achieve a subconfluence. Scraping cells with a sharp tip vertically to the marked lines and washing with PBS, a cell-free area was made. Cells migrating into the scraped areas were counted after 0, 6, 12, and 24 h, respectively.

### Statistical analyses

Results are expressed as the mean ± standard error of the mean (SEM). Student’s *t* test or one-way ANOVA test was used for statistical analysis performed using SPSS version 16.0. *P* < 0.05 was considered to be statistically significant.

## Results

### HMGB1 and RAGE are expressed highly in HCCLM3 cells

**Fig. 1:**
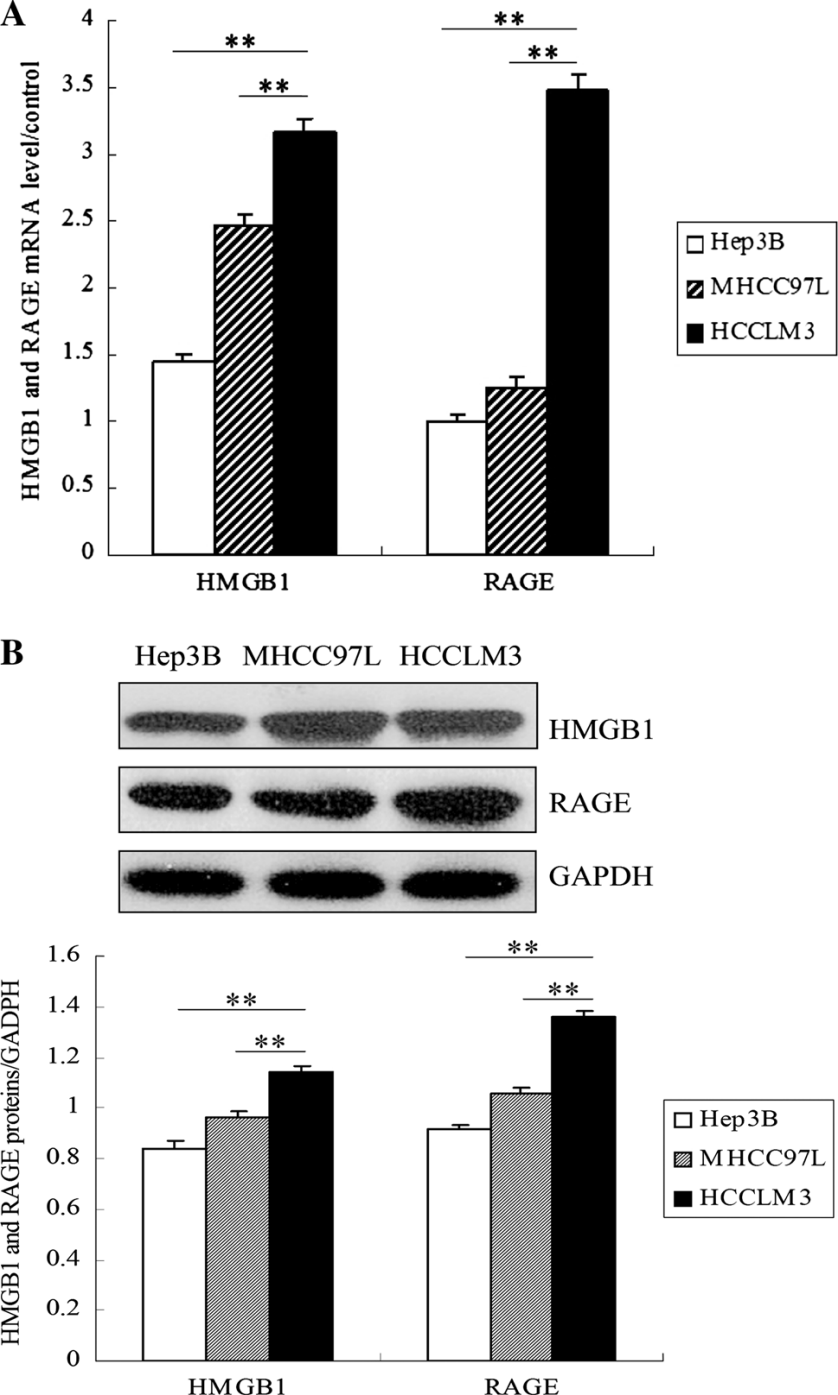
HMGB1 and RAGE were expressed highly in HCCLM3 cells. **a** Relative expression of HMGB1 or RAGE mRNA normalized to β-actin was measured by qRT-PCR in Hep3B, MHCC97L, and HCCLM3 cells. **b** Expression of HMGB1 or RAGE protein in the three HCC cell lines was examined by Western blot. GAPDH was used as an internal control for quantity analysis. (**P < 0.01, *P < 0.05)

To study the role of HMGB1 and RAGE in HCC cells, we first examined the expressions of HMGB1 and RAGE in HCC cells with distinct metastasis potentials by RT-PCR and western blot. All three HCC cells expressed HMGB1 and RAGE. Compared with Hep3B and MHCC97L cells, HCCLM3 cells expressed the highest HMGB1 and RAGE mRNA (Fig. 1a). Concomitantly, HMGB1 and RAGE proteins were significantly increased in HCCLM3 cells (Fig. 1b). These results indicate that the expressions of HMGB1 and RAGE mRNA and proteins are related to the metastasis potentials of HCC cells.

### Extracellular HMGB1 and sRAGE are increased in HCCLM3 cells

**Fig. 2:**
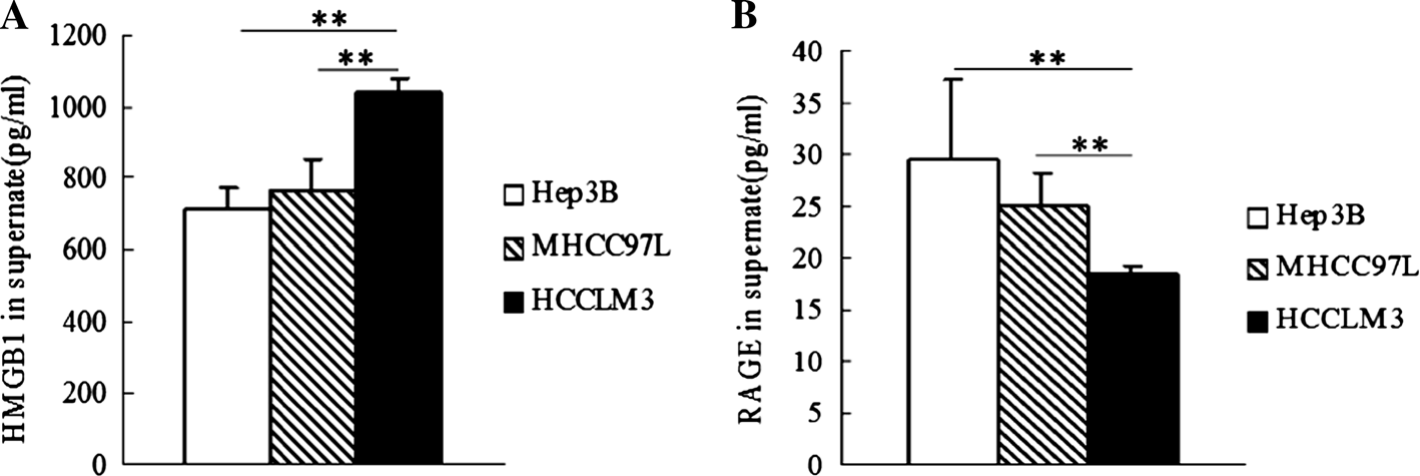
Extracellular HMGB1, and sRAGE level in the supernatants of HCC cells. The release level of extracellular HMGB1 (**a**) and sRAGE (**b**) was measured by ELISA in the supernatants of Hep3B, MHCC97L, and HCCLM3 cells. (*P < 0.05, **P < 0.01)

ELISA results showed that HMGB1 was released the highest in HCCLM3 cells (1041.667 ± 39.425 pg/ml) than that in Hep3B cells (709.667 ± 62.581 pg/ml) (*P* < 0.01), or MHCC97L cells, respectively (761.667 ± 96.407 pg/ml) (*P* < 0.01) (Fig. 2a). The concentration of sRAGE in the supernatants collected from HCCLM3 cells (22.520 ± 0.935 pg/ml) was less than that from Hep3B cells (31.903 ± 2.984 pg/ml) (*P* < 0.05), or MHCC97L cells (24.966 ± 3.212 pg/ml) (*P* < 0.05), respectively (Fig. 2b). It is suggested that the release level of HMGB1 or sRAGE extracellularly is associated with the metastasis potentials of HCC cells.

### HMGB1 antibody, RAGE antibody, or sRAGE attenuates survival rate of HCCLM3 cells, while HMGB1 increases it

**Fig. 3:**
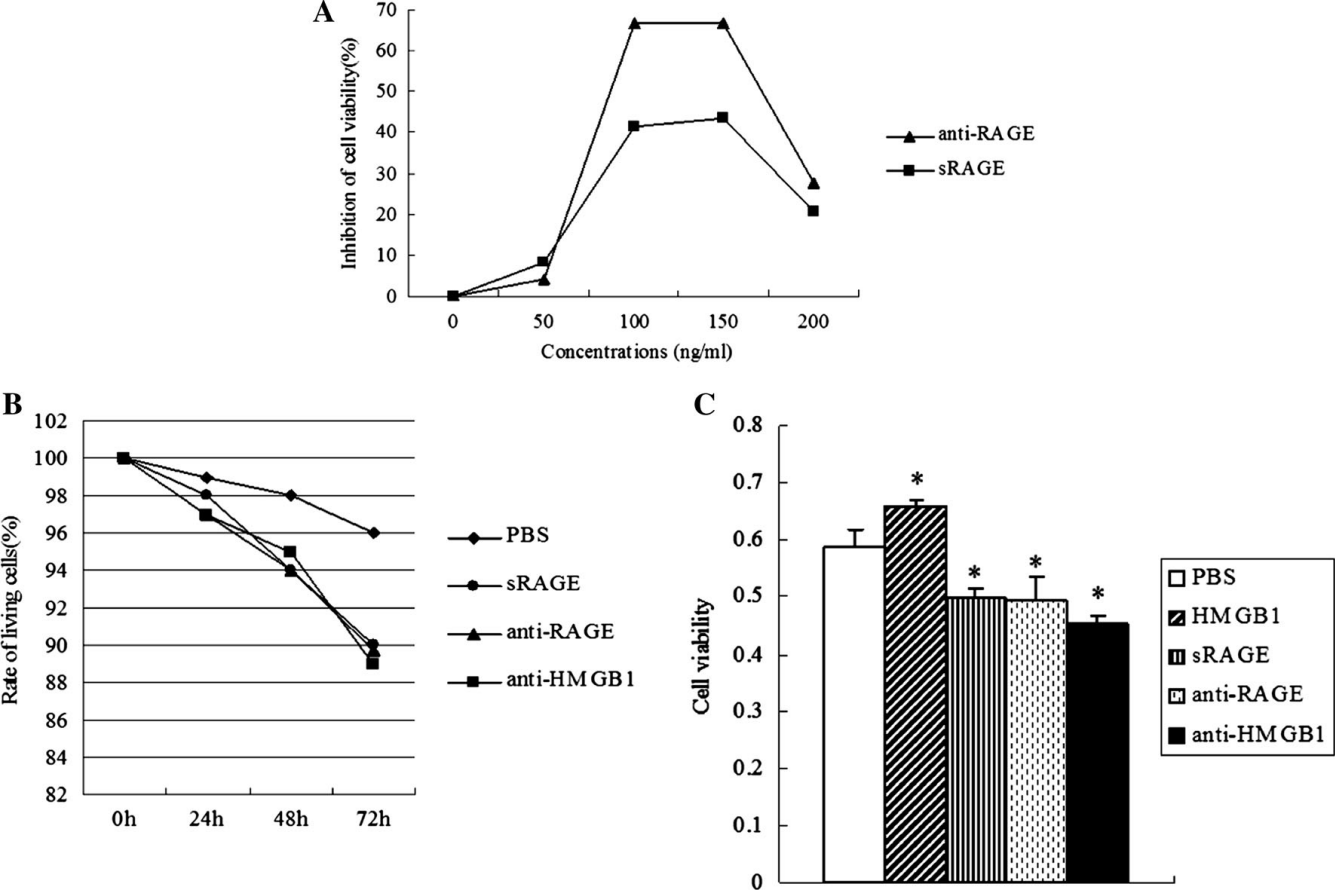
Effects of HMGB1 or RAGE antibodies, sRAGE on the survival rate of HCCLM3 cells. **a** Cell viability of HCCLM3 cells was analyzed by MTT after interacting with anti-RAGE neutralizing antibody, or human recombinant sRAGE at various concentrations (50, 100, 150, and 200 ng/ml), respectively for 24 h. RAGE antibody or sRAGE reduced the cell viability of HCCLM3 cells dose dependently, and both achieved the greatest growth inhibition at 150 ng/ml. **b** HCCLM3 cells were treated with anti-HMGB1 neutralizing antibody (20 ng/ml), anti-RAGE neutralizing antibody (150 ng/ml), and sRAGE (150 ng/ml), respectively for 24–72 h. Living rate of HCCLM3 cells was decreased by HMGB1 or RAGE antibody and sRAGE after incubating for 24 h and reached the greatest surviving inhibition after 72 h. **c** HCCLM3 cells were incubated with rhHMGB1 (50 ng/ml) for 48 h, then MTT assay showed that HMGB1 increased the living rate of HCCLM3 cells. (*P < 0.05, **P < 0.01)

To explore the role of the HMGB1-RAGE axis on the cell viability of HCC cells, HCCLM3 cells were treated with anti-HMGB1 neutralizing antibody, anti-RAGE neutralizing antibody, exogenous sRAGE protein, and recombinant human HMGB1(rhHMGB1), respectively. The work concentrations of recombinant human HMGB1 and anti-HMGB1 neutralizing antibody were 50 and 20 ng/ml, respectively. HCCLM3 cells were incubated for 24 h with varying concentrations (50, 100, 150, and 200 ng/ml) of anti-RAGE neutralizing antibody or sRAGE, respectively. MTT assay results showed that anti-RAGE neutralizing antibody and sRAGE significantly reduced the cell viability of HCCLM3 cells in a concentration-dependent manner, and both reached a peak at 150 ng/ml (Fig. 3a). Thus, the optimal work concentrations of anti-RAGE neutralizing antibody and sRAGE were determined as 150 ng/ml.

The results showed that anti-RAGE neutralizing antibody, anti-HMGB1 neutralizing antibody, and sRAGE started to reduce the living rate of HCCLM3 cells from 24 h and reached a lowest level at 72 h by the trypan blue exclusion assay (Fig. 3b). MTT assay also showed that the cell viability of HCCLM3 cells was significantly attenuated by anti-RAGE neutralizing antibody, anti-HMGB1 neutralizing antibody, and sRAGE, while increased by rhHMGB1 at 48 h (Fig. 3c). These findings indicated that blockade of HMGB1 and RAGE interaction correlates with the survival of HCC cells.

### HMGB1 siRNA and RAGE siRNA decrease cell viability of HCCLM3 cells

**Fig. 4:**
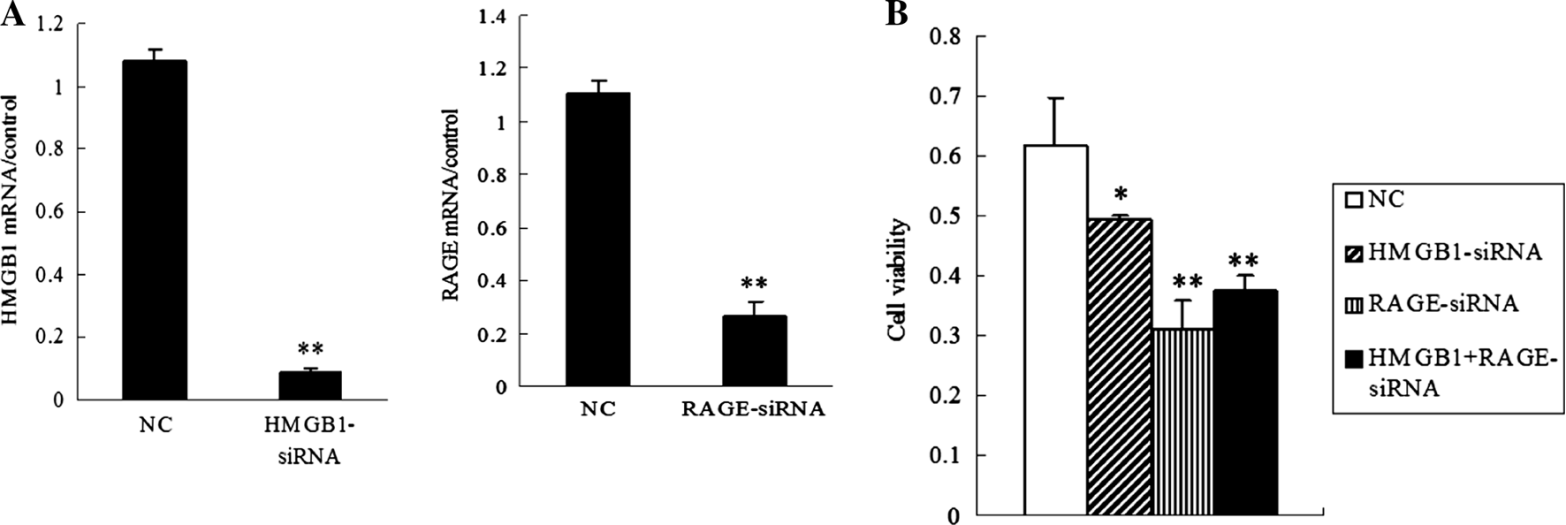
HMGB1 siRNA and RAGE siRNA decreased the cell viability of HCCLM3 cells. **a** Knockdown of HMGB1 or RAGE in HCCLM3 cells by specific gene-targeted HMGB1-siRNA and RAGE-siRNA was confirmed by qRT-PCR. Add a descriptive label of the figure here. **b** After incubating with HMGB1-siRNA (75 ng/ml), RAGE-siRNA (75 ng/ml), and rhHMGB1 (50 ng/ml) for 48 h, the results of MTT assay showed that knockdown of HMGB1 or RAGE decreased the cell viability of HCCLM3 cells (*P < 0.05, **P < 0.01). *NC* negative control with a nonsense siRNA sequence

To test the hypothesis that HMGB1 and RAGE expressions promote HCC cells to survive, we then defined specific siRNAs-targeted HMGB1 gene and RAGE gene, respectively, which were instantly transfected into HCCLM3 cells. HMGB1 mRNA and RAGE mRNA expressions were significantly decreased by specific HMGB1-siRNA and RAGE-siRNA, respectively (Fig. 4a). The results showed that targeted knockdown of HMGB1 or RAGE decreased the cell viability of HCCLM3 cells, which was not retrieved by adding ectogenic rhHMGB1 (Fig. 4b).

### Invasion and mobility activity of HCCLM3 cells were inhibited by HMGB1/RAGE siRNA or antibody

**Fig. 5:**
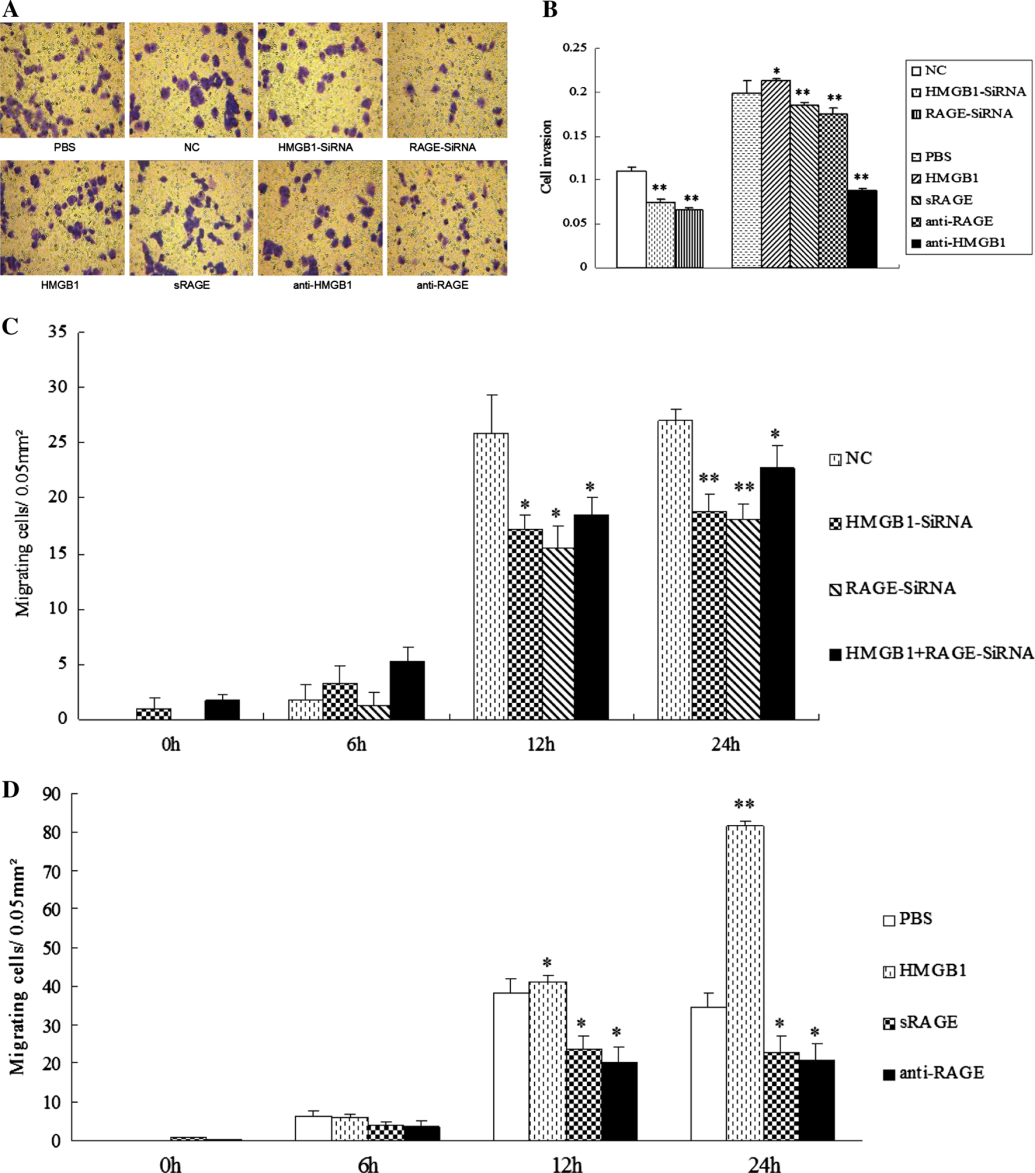
HMGB1 siRNA and RAGE siRNA attenuated invasion and mobility of HCCLM3 cells in vitro. HCCLM3 cells were seeded into the upper chamber of the transwell, treated with HMGB1-siRNA, RAGE-siRNA, anti-RAGE antibody or sRAGE, and rhHMGB1, and allowed to invade matrigel for 24 h. **a** The invasive cells migrating through the basal membrane to its lower surface were stained with crystal violet, then were photographed (20 × 10). **b** The number of invasive cells was also quantified by dissolving the purple crystals on the membranes in 500 μl 10 % acetic acid, and measuring their OD values at 570 nm by Multiskan Ascent. Cell invasion ability was expressed indirectly by varying OD values. HMGB1 or RAGE siRNA, HMGB1, or RAGE antibody, and sRAGE inhibited the invasion ability of HCCLM3 cells, while rhHMGB1 facilitated it (*P < 0.05, **P < 0.01). **c**, **d** Migration ability of HCCLM3 cells was detected by wound healing assay. Incubating for 0, 6, 12, and 24 h, respectively, the number of HCCLM3 cells migrating into the scraped areas was counted. (*P < 0.05, **P < 0.01)

Transwell assay showed that knockdown of HMGB1 and RAGE evidently reduced the cell invasive ability of HCCLM3 cells, respectively. We also observed that treatments with anti-HMGB1 antibody, anti-RAGE antibody, or sRAGE significantly decreased the invasion of HCCLM3 cells, while rhHMGB1 actively facilitated it (Fig. 5a, b). Consistent with these findings, HCCLM3 cells treated with HMGB1 siRNA, RAGE siRNA, anti-RAGE neutralizing antibody, and sRAGE, respectively, displayed a considerably decrease in the cell mobility at 12 and 24 h (Fig. 5c), while HMGB1 obviously promoted cell mobility at 24 h (Fig. 5d). Moreover, the effect of HMGB1 on cell mobility was abolished by RAGE-siRNA (Fig. 5c), indicating that HMGB1 promotes the mobility of HCCLM3 cells in a RAGE-dependent way.

### HMGB1 siRNA and RAGE siRNA decrease the expressions of NF-κB p50 and p65

**Fig. 6:**
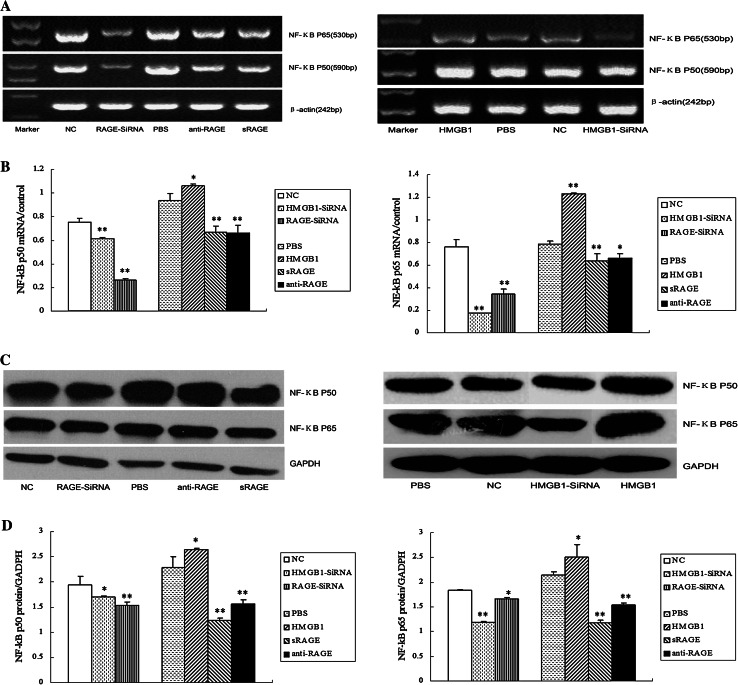
Effects of HMGB1 and RAGE on NF-κB expression in HCCLM3 cells. **a** Expression of NF-κB p65 or p50 mRNA in HCCLM3 cells was explored by RT-PCR. **b** Relative expression of NF-κB p65 or p50 mRNA was normalized to β-actin. HMGB1 or RAGE siRNA, HMGB1 or RAGE antibody, and sRAGE inhibited NF-κB p65 and p50 mRNA expression in HCCLM3 cells, while rhHMGB1 increased it (* *P* < 0.05, ** *P* < 0.01). **c** Western blot was performed to test NF-κB p65 or p50 protein expression in HCCLM3 cells. **d** Quantity analysis of NF-κB p65 and p50 proteins expression levels relative to GAPDH. (* *P* < 0.05, ** *P* < 0.01)

Emerging studies have suggested that NF-κB-signaling pathway contributes to RAGE-driven carcinogenesis. To explore the effect of HMGB1-RAGE axis on NF-κB expression, siRNA or antibodies was used to interfere with the HMGB1-RAGE interaction, which was activated by exogenous rhHMGB1. Knockdown of HMGB1 or RAGE inhibited NF-κB p50 and p65 mRNA expressions in HCCLM3 cells, respectively, and we also observed that NF-κB p50 and p65 mRNA expressions were decreased by intervention with anti-RAGE neutralizing antibody or sRAGE. In contrast, HMGB1 slightly increased them (Fig. 6a, b). The results of NF-κB p50 and p65 proteins are concomitant with these findings (Fig. 6c, d).

## Discussion

Inflammation facilitates the occurrence and development of tumors. The biologic effects of local inflammation environment, also known as “tumor environment,” are to maintain proliferative signals, promote angiogenesis, and boost cell invasion and metastasis [22]. HMGB1 is constitutively expressed in the nucleus of cells, and also can be released outside by tumor cells and by inflammatory cells [23]. In the nucleus, as a DNA chaperone, it mediates different functions such as DNA repair and recombination, transcription, and stabilization of nucleosomes [24]. While extracellular HMGB1 also participates in many biological processes, such as immunomodulatory role of sepsis or noninfectious inflammation, angiogenesis, wound healing, and tumorigenesis [25]. Constant release of HMGB1 outside the cells, as a damage-associated molecular pattern (DAMP), creates a tumor microenvironment, which contributes to the development of epithelial malignancies [26].

Emerging evidences have demonstrated that chronic infection of HBV or HCV can induce the translocation of HMGB1 from nuclei to cytoplasm and extracellular release of HMGB1 [4, 27]. Serum HMGB1 levels were found significantly higher in HCC patients and overexpression of HMGB1 was associated with their clinico-pathological features and outcomes [28]. Our group found that HMGB1 and RAGE can modulate the proliferation and cell cycle of HCC cells [10]. These findings show that HMGB1-RAGE axis may play an important role in the development of HCC, such as the migration and invasion of HCC. In order to elucidate that, three HCC cell lines with different metastasis potentials were analyzed in our study. The expressions of HMGB1 and RAGE increased consistently with the metastasis potentials of HCC cell lines, indicating that HMGB1 and RAGE are associated with metastasis potential of HCC cells. Then, HCCLM3 cell line was selected for further study, for its highest metastasis potential and richest expressions of HMGB1 and RAGE in all three cell lines. The results showed RAGE-dependent stimulating responses induced by rhHMGB1 including increased proliferation, mobility, and invasion of HCCLM3 cells, whereas down regulation of the endogenous expression of HMGB1/RAGE by RNAi technology or administration of anti-HMGB1/anti-RAGE antibodies remarkably attenuated these effects. This finding illustrated that both HMGB1 and RAGE were essential factors in the proliferation, migration, and invasion of HCC cells, with a HMGB1-RAGE interaction mode. The results also suggested that the HCCLM3 cells with weakened viability showed low ability of invasiveness and motility. Reduced viability of HCC cells is likely contributed to the decline of cell motility and invasiveness. We also found that the concentration of HMGB1 in the supernatants of HCCLM3 cells was much higher compared to that in low-metastatic MHCC97L cells and nonmetastatic Hep3B cells. Regarding that cell viability was not obviously reduced at that time point, it suggested that HMGB1 can be actively secreted into extracellular environment by HCC cells, which, thereby participated in the construction of “tumor microenvironment.”

Soluble form of RAGE is the major isoform of RAGE, mutated from the shedding of extracellular domain of RAGE or RAGE mRNA selectively splicing (the latter also known as endogenous secretory RAGE). Due to the absence of intracellular domain and membrane mosaic area of the receptor, sRAGE is deprived of the ability of signaling transduction. Therefore, when HMGB1 combines with decayed sRAGE, the ability of interacting with complete RAGE on the cell membrane is attenuated, which subsequently inhibits its important biological behavior of tumorigenesis promotion [29, 30]. Studies showed that, compared to healthy volunteers, the level of sRAGE in serum was lower than that from patients of breast cancer [31] and gastric cancer [32]. Similarly, in this study, we found that the concentration of sRAGE is negatively correlated with the metastatic potentials of HCC cell lines. Meanwhile, administration of exogenous sRAGE decreased the cell viability, invasion, and mobility, indicating that sRAGE may become a potential therapeutic target of HCC. However, more evidences from experiments in vivo and vitro were required. Interestingly, we also found that knockdown of RAGE showed more apparent decrease of cell viability, invasion, and mobility than that of HMGB1, suggesting that other soluble RAGE ligands of RAGE such as S100, AGEs may also be likely involved in this process. Soluble RAGE demonstrated by Kalea et al. [33], acts to inhibit cancer cells by activation of MAPK families, which may play an important role in tumorigenesis and the development of HCC. Whether soluble RAGE inhibits HCC cells through activating MAPK-signaling pathway or by other mechanisms remains unclear, and needs to be elucidated in our future studies.

NF-κB, as a quick responsive transcription factor, is the converging point of various signaling pathways. In inflammation-related tumor model of animals, the IKKβ-dependent activation of NF-κB is the key process in inflammation-induced carcinoma. Therefore, we postulated that the NF-κB-signaling pathway may be engaged in the biological function of HMGB1-RAGE axis in HCC development. Our results showed that rhHMGB1 increased the expression of NF-κB p50 and p65. Conversely, knockdown of HMGB1 and RAGE specifically by siRNA significantly reduced the level of NF-κB p50 and p65. Impacts of HMGB1-RAGE axis on activation of NF-κB-signaling pathway and its downstream transcriptional activity in HCC are worthy of being determined in our future work.

In summary, we demonstrate that HMGB1-RAGE axis plays a pivotal role in HCC cell invasion and migration. The expression alteration of NF-κB is also involved in this process. However, many issues remain unclear: what are the downstream signaling pathways of HMGB1-RAGE axis in the development of HCC? Whether NF-κB is activated in this process or not? Further explorations, especially in vivo studies are needed to confirm them.

## Abbreviations

HMGB1: High mobility group protein box1
HCC: Hepatocellular carcinoma
NF-κB: Nuclear factor kappa B
RAGE: Receptor for advanced glycation end products
sRAGE: Soluble form of RAGE
rhHMGB1: Recombinant human HMGB1
